# Characterization of a Near Full-Length Hepatitis E Virus Genome of Subtype 3c Generated from Naturally Infected South African Backyard Pigs

**DOI:** 10.3390/pathogens11091030

**Published:** 2022-09-11

**Authors:** Ravendra P. Chauhan, Michelle L. Gordon

**Affiliations:** School of Laboratory Medicine and Medical Sciences, College of Health Sciences, University of KwaZulu-Natal, Durban 4001, South Africa

**Keywords:** backyard pig farm, hepatitis E virus, HEV-3c, HEV zoonosis, Illumina sequencing, phylogenetic analysis, *Sus scrofa domesticus*

## Abstract

Eight genotypes of the hepatitis E virus (*Orthohepevirus* *A*; HEV) designated HEV-1 to HEV-8 have been reported from various mammalian hosts. Notably, domestic pigs and wild boars are the natural reservoirs of HEV-3 and HEV-4 genotypes with zoonotic propensity. Since HEV infection in domestic pigs is usually subclinical, it may remain undetected, facilitating zoonotic spillover of HEV to the exposed human populations. A previous study from our group in 2021, using deep sequencing of a pooled saliva sample, generated various swine enteric virus genomes, including a near full-length swine HEV genome (7040 nt; 97.7% genome coverage) from five-month-old grower pigs at a backyard pig farm in the uMgungundlovu District, KwaZulu-Natal, South Africa. In the present study, we describe the further characterization, including genotyping and subtyping of the swine HEV isolate using phylogenetics and ‘HEVnet Typing Tool’. Our analyses confirmed that the South African swine HEV genome characterized in this study belonged to HEV genotype 3 subtype 3c (HEV-3c). While HEV-3c infections in domestic pigs have been previously reported from Brazil, Germany, Italy, and the Netherlands, they only generated partial genome sequences of open reading frame 1 (ORF1) and/or ORF2. To our knowledge, this is the first near full-length swine HEV-3c genome generated from naturally infected domestic pigs (*Sus scrofa domesticus*) in South Africa. However, due to the gap in the information on the HEV-3c genome sequences in various geographical locations worldwide, including South Africa, the epidemiology of the South African swine HEV genome characterized in this study remains inconclusive. Molecular and genomic surveillance of HEV in domestic pig populations in South Africa would be useful to determine their prevalence, circulating subtypes, and zoonosis risk.

## 1. Introduction

Swine or domestic pigs (*Sus scrofa domesticus*) are the reservoirs of several virus pathogens [1,2,3,4,5,6,7,8]. Due to subclinical infection, the prevalence of some of these viruses in domestic pigs, such as the hepatitis E virus (*Orthohepevirus A*; HEV), may remain undetected and thus underestimated [9]. Eight genotypes of HEV designated HEV-1 to HEV-8 have been reported in various mammalian hosts [10,11,12,13,14,15]. The first report of HEV causing natural infections in domestic pigs appeared from a commercial pig farm in the Midwestern United States in 1997 [16]. Subsequently, numerous reports of HEV infections in domestic pigs have appeared worldwide, suggesting that domestic pigs are the natural reservoirs of HEV genotype 3 (HEV-3) and HEV-4 [17]. 

Various experimental studies have demonstrated that pig-to-pig transmission of HEV can occur through contact on pig farms [18,19,20]; however, pig-to-human HEV transmission is suggested to have occurred due to the consumption of contaminated pork meat [21]. A systematic review and meta-analysis by Li et al. (2020) reported an overall 12.47% anti-HEV IgG and 1.47% anti-HEV IgM seroprevalence in the tested human populations worldwide. In addition, they reported an overall 0.2% molecular prevalence (HEV RNA) in tested human populations globally, confirming active HEV infections [22]. While HEV infection is usually acute self-limiting or asymptomatic in immunocompetent individuals [23], the immunocompromised individuals or those with existing medical conditions, such as transplant recipients [24], patients with existing liver disease [25], or cancer [26,27] may develop chronic hepatitis due to HEV infection [8].

Numerous studies have suggested foodborne transmission of HEV to humans and have reported the prevalence of HEV RNA in porcine liver and pork products from different countries. For example, Fu et al. (2010) generated partial HEV genomes from a domestic pig and a human originating from the same district in China, which had 100% identical nucleotide sequences and therefore suggested the possibility of foodborne HEV transmission to the human population [28]. Salines et al. (2017) reported the prevalence of HEV RNA in pig liver samples (0.8 to 20.8%; *n* = 10 studies) and in retail pork products (0 to 58.0%; *n* = 9 studies) in different countries [29]. In addition, Silva et al. (2018) reported a high HEV seroprevalence (77.6% in 2012 and 65.5% in 2014) and a 0.8% prevalence of HEV RNA in tested Brazilian backyard pigs. They reported that the partial swine HEV genomes generated were similar to human HEV strains reported in Brazil [30]. Another study from Southern Brazil reported a 20% prevalence of HEV RNA in pig fecal samples collected from a small-scale farm in 2014. The partial swine HEV genomes generated were closely related to the HEV genomes reported earlier from the human populations in Brazil [31]. Recently, Li et al. (2022) reported an overall 60% HEV seroprevalence in tested domestic pigs worldwide; however, the prevalence of HEV RNA in tested domestic pigs and pork products was 13% and 10%, respectively [32]. The available data suggested that the prevalence of HEV RNA in the porcine liver [33,34,35,36,37,38,39] and pork meat samples [40,41,42,43] pose a potential risk of foodborne transmission to humans [21,44], through the consumption of raw or undercooked pork meat [35,45]. 

While currently, there is no evidence of direct pig-to-human HEV transmission through contact, various studies have suggested the occupation-related exposure of HEV to humans. For example, Hoan et al. (2019) reported HEV-3 RNA in 12.4% of domestic pig liver tissue samples tested in Hanoi, Viet Nam. They reported a significantly higher HEV seroprevalence in slaughterhouse workers, pig farmers, and pork meat vendors than the unexposed individuals [46]. Similarly, while HEV-4 RNA was detected in young domestic pigs in Beijing, China, a significantly higher HEV seroprevalence was detected in farm workers and slaughterhouse workers than in the general public [47]. Several other studies have also reported a high HEV seroprevalence in occupationally exposed human populations, such as pig farm workers in Moldova [48], Israel [49], India [50], and pig farm workers and veterinarians in France [51].

In the African continent, a recent study by Bagulo et al. (2022) reported HEV seroprevalence in domestic pigs in Ghana [52]. Previously, Modiyinji et al. (2020) reported HEV seroprevalence and HEV-3 RNA in the feces of domestic pigs in Cameroon [53]. Owolodun et al. (2014) reported a high overall prevalence of HEV-3 RNA (76.7%) and HEV seroprevalence (55.6%) in tested Nigerian domestic pigs [54]. In South Africa, Adelabu et al. (2017) reported a 4.4% prevalence of HEV RNA in pig feces in commercial and communal pig farms in the Eastern Cape province [55]. They generated seven partial HEV-3 capsid protein sequences ranging from 388 to 427 nucleotides (GenBank accessions: KX896664–KX896670) [55]. Of note, there was no report of HEV in South African backyard pig populations. In a previous study in 2021, using metagenomics, we, for the first time, obtained numerous swine enteric RNA virus genomes from the saliva of the backyard pigs in the uMgungundlovu District of the KwaZulu-Natal province, South Africa [56]. We obtained a near full-length swine HEV genome, which remained uncharacterized. In the present study, we characterized the near full-length swine HEV genome generated from South African backyard pigs.

## 2. Materials and Methods

### 2.1. Ethics Approval

Full approval of the research protocol was obtained from the Animal Research Ethics Committee of the University of KwaZulu-Natal; Reference# AREC/041/019D. We obtained the Section 20 permit from the Department of Agriculture, Land Reform and Rural Development (DALRRD), South Africa, in terms of the Animal Diseases Act, 1984 (Act No. 35 of 1984); Reference# 12/11/1/5/4 (1425 AC) (1).

### 2.2. Study Design

A previous study performed passive surveillance for detecting influenza A virus (IAV) RNA in backyard pig oral secretion (saliva) samples (*n* = 102) collected in March 2021 from three backyard pig farms in the uMgungundlovu District of the KwaZulu-Natal province, South Africa. Using a TaqMan probe-based one-step real-time RT-PCR assay that detects IAV matrix gene, we did not detect IAV RNA [57]. Intriguingly, some of the pigs sampled exhibited clinical signs of disease, such as red patches on the skin. This prompted us to investigate the oral RNA virome of one of the pooled saliva samples obtained from five-month-old grower pigs exhibiting clinical signs of disease. We used deep sequencing to determine the diversity of oral RNA virome of the selected saliva sample. While we generated the genomes of multiple swine enteric viruses [56], a near full-length genome of swine HEV was also generated, which remained uncharacterized and therefore was analyzed in the present study.

### 2.3. Sample Collection and Processing

After obtaining written informed consent confirming the voluntary participation of the backyard pig farmers, the backyard pigs were visually screened for clinical signs of illness with the assistance of an Animal Health Technician (AHT) from the State Veterinary Department. For the present study, we chose a pooled saliva sample taken from five-month-old growers with red patches on the skin to determine their oral RNA virome. The pig saliva was collected using a standard non-invasive hanging rope method [58,59]. Briefly, about 80 cm long three-strand twisted 100% cotton rope was suspended in the air inside the pen; the confined pigs were allowed to chew the rope for about 15 min. While seven growers simultaneously chewed the rope, most of them had red patches on their skin. The rope was squeezed in a plastic Ziploc bag, and the saliva was aseptically collected into a 15 mL Falcon tube and transported to a biosafety level-2 (BSL-2) laboratory at the University of KwaZulu-Natal on dry ice. The saliva was centrifuged at 1500× *g* for 10 min at 4 °C to eliminate the feed contaminants, aliquoted into 2 mL sterile cryovials, and stored at −80 °C for downstream processing. One aliquot containing 500 μL of saliva was shipped on dry ice to the Biotechnology Platform Laboratory of the Agricultural Research Council, Onderstepoort, Pretoria, for deep sequencing.

### 2.4. Metagenomic Sequencing

The viral RNA was extracted using the NucleoMag Pathogen kit (Macherey-Nagel, Dueren, Germany). The ribosomal RNA was depleted, and the library preparation was performed using the Illumina Stranded Total RNA Prep (Catalogue# 20040529) according to the manufacturer’s specifications. Deep sequencing was performed on an Illumina HiSeq X instrument (San Diego, CA, USA).

### 2.5. Genome Assembly and Annotation

The FASTQ files containing 125 bp paired-end reads were analyzed using Genome Detective v 1.135 (www.genomedetective.com; accessed on 7 September 2021) [60], an integrated platform to perform quality control. Briefly, the backyard pig saliva sample generated 7,953,568 raw reads. The Genome Detective performed quality control and trimmed the raw reads, filtered out low-quality reads and adapters (1,565,304; 19.7%), and non-viral reads (6,212,686; 78.1%). The remaining reads (175,578; 2.2%) were processed for virus contig de novo assembly. The Genome Detective verified the resulting contigs using reference genomes available in the NCBI database to determine percent genome coverage and percent nucleotide and amino acid identities of the assembled genomes. The HEV genome generated resulted in 97.7% genome coverage. We then manually performed nucleotide BLAST analysis to verify the percent nucleotide identity of the South African swine HEV genome with other HEV genomes available in NCBI-GenBank. In addition, we analyzed the swine HEV genome characterized in the present study with HEV reference genomes to determine insertions and deletions using ‘Geneious Prime 2021.2.2’ (Biomatters, Auckland, New Zealand). Later, we performed the multiple sequence alignment of the South African swine HEV genome with various full-length HEV-1 to HEV-8 genotypes to determine the stop codons in the South African swine HEV genome in ‘Geneious Prime 2022.0.1’ (Biomatters, Auckland, New Zealand).

### 2.6. Phylogenetic Analysis and ‘HEVnet Typing Tool’ Analysis

We performed a detailed phylogenetic analysis to characterize the generated swine HEV genome. Briefly, to determine the swine HEV genotype, we downloaded full-length HEV-1 to HEV-8 genome sequences from the NCBI-GenBank database, reported from various hosts. We identified the best substitution model for the phylogenetic analysis using ‘MEGA-X’ [61]. A phylogenetic tree was drawn using PhyML [62] with the GTR+G+I model of substitution and 1000 bootstrap replications in ‘Geneious Prime 2022.0.1′. We then used the ‘HEVnet Typing Tool’ (https://www.rivm.nl/mpf/typingtool/hev/, accessed on 11 January 2022) [63] to identify the subtype of the South African swine HEV genome. For the confirmation of the HEV subtype, a PhyML phylogenetic tree of full-length genome sequences of various HEV-3 subtypes was constructed using the GTR+G+I model of substitution with 1000 bootstrap replications. To determine the epidemiology of the South African swine HEV-3c genome, we constructed a PhyML tree of full-length HEV-3c genomes reported from various hosts worldwide using the GTR+G+I model of substitution with 1000 bootstrap replications.

## 3. Results

**Figure 1:**
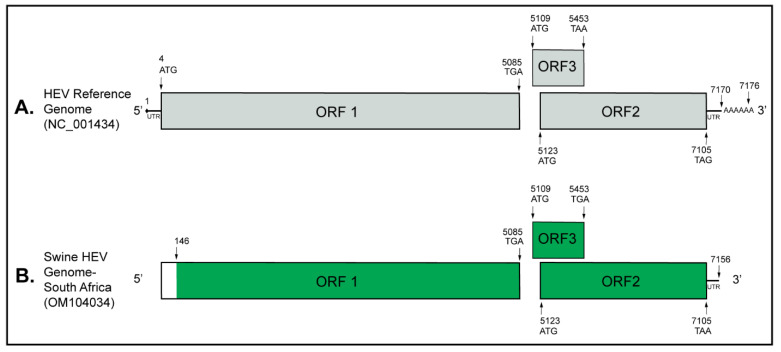
Schematic representation of the swine HEV genome characterized in this study. (**A**) The HEV reference genome (GenBank accession: NC_001434) comprises a 5′ 7-methylguanosine cap followed by a short untranslated region (UTR). It has a 3′ UTR and a polyadenylated tail. (**B**) The South African swine HEV genome characterized in the present study (7040 nt; GenBank accession: OM104034) extends from the nucleotide position 146 at the 5′ end to 7156 at the 3′ end compared to the reference genome. The South African swine HEV genome comprises 97.7% genome coverage (represented in green color) having complete coding sequences for the ORF2 and ORF3 and a partial coding sequence for the ORF1.

The Genome Detective determined 74.3% nucleotide and 84.8% amino acid identity of the generated near full-length South African swine HEV genome (7040 nt; GenBank accession: OM104034) with the NCBI-designated HEV reference genome representing the complete HEV genome (GenBank accession: NC_001434). In addition, the South African swine HEV genome generated shared 89.2% nucleotide and 96.7% amino acid identity with the HEV-3c reference genome designated by Smith et al. (2020) (GenBank accession: FJ705359) [64]. The near full-length HEV genome (7040 nt; 97.7% genome coverage) generated from the South African backyard pig saliva had three stop codons, TGA, TGA, and TAA, at nucleotide positions 5085, 5453, and 7105, respectively, resulting in three open reading frames (ORFs): ORF1, ORF3, and ORF2, respectively. While only a partial coding sequence was generated for ORF1, the complete coding sequences were generated for ORF2 and ORF3 (Figure 1). 

Using multiple sequence alignment of several HEV genotypes reported from various hosts in different geographical locations, we determined that the stop codon for ORF1 (TGA) was conserved in all HEV genotypes. The stop codon for ORF2 in all HEV-4 genomes was TGA, while HEV-1 and HEV-2 had TAG, and all other HEV genotypes, i.e., HEV-3 and HEV-5 to HEV-8 had TAA. However, the stop codon for ORF3 was less conserved among the genotypes, varying between TGA and TAA. This variation in the stop codons in HEV genomes may be due to natural selection during the long-term co-evolution of HEV in their respective hosts [65,66].

**Figure 2:**
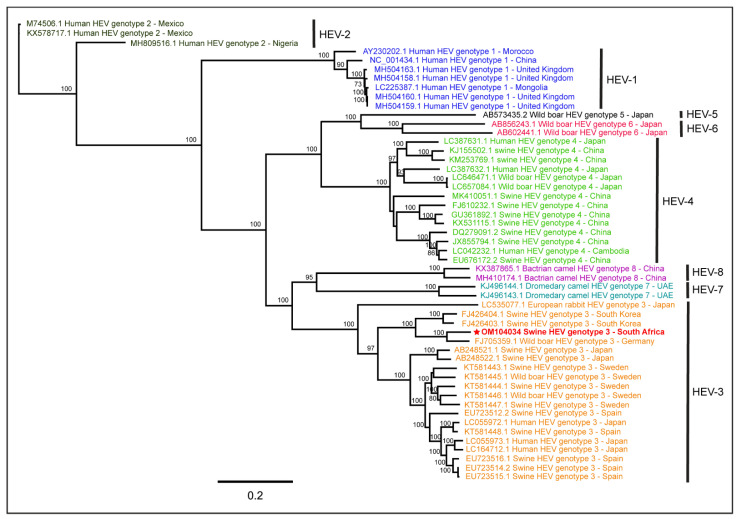
Molecular characterization of the swine hepatitis E virus (HEV) genome generated from the backyard pig saliva in South Africa. The PhyML tree of the full-length HEV-1 to HEV-8 genomes reported from various hosts worldwide determined that the South African swine HEV genome belonged to HEV genotype 3 (HEV-3).

The PhyML tree (Figure 2) of the full-length HEV-1 to HEV-8 genomes reported from various hosts worldwide showed that the swine HEV genome analyzed in this study was more closely related to the HEV genotype 3 (HEV-3) genome reported from a wild boar in Germany (GenBank accession: FJ705359). The nucleotide BLAST analysis and pairwise sequence alignment showed that the generated South African swine HEV genome shared 89.2% pairwise nucleotide identity with the HEV-3 genome reported from the wild boar in Germany (GenBank accession: FJ705359). This observation confirmed that the swine HEV genome generated from the South African backyard pig saliva belonged to the HEV-3 genotype. 

**Figure 3:**
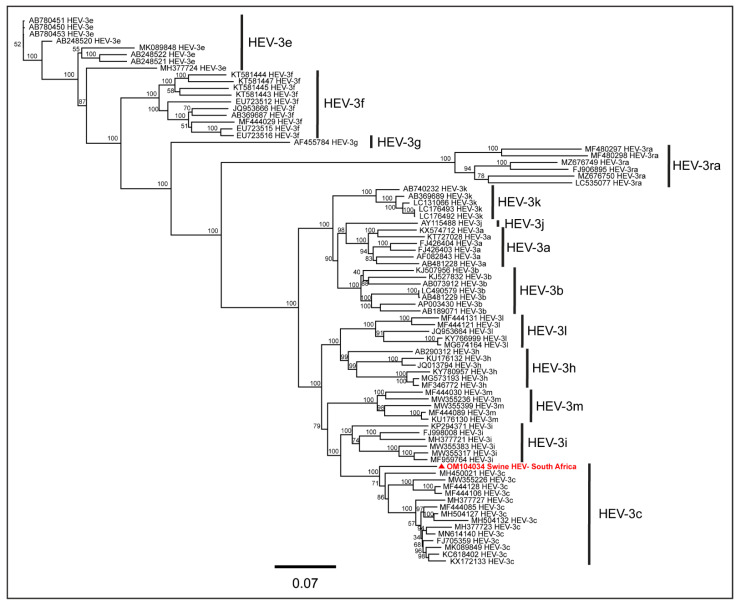
The PhyML tree of full-length genomes of various HEV-3 subtypes available at NCBI-GenBank and the South African swine HEV genome (highlighted in red). The South African isolate clustered with the HEV-3c genomes reported from humans and wild boars in different countries, thus confirming that the South African isolate belonged to the HEV-3c subtype.

The subtype analysis using ‘HEVnet Typing Tool’ [63], an integrated HEV genotyping and subtyping tool, showed that the South African swine HEV genome belonged to the HEV-3c subtype. To confirm this, we conducted phylogenetic analysis using the full-length genomes of various HEV-3 subtypes available in the NCBI-GenBank reported globally. As shown in the PhyML tree (Figure 3), the South African swine HEV genome clustered with the HEV-3c genomes reported from humans and wild boars in different countries, confirming that the swine HEV genome generated in the present study belonged to the HEV-3c subtype.

**Figure 4:**
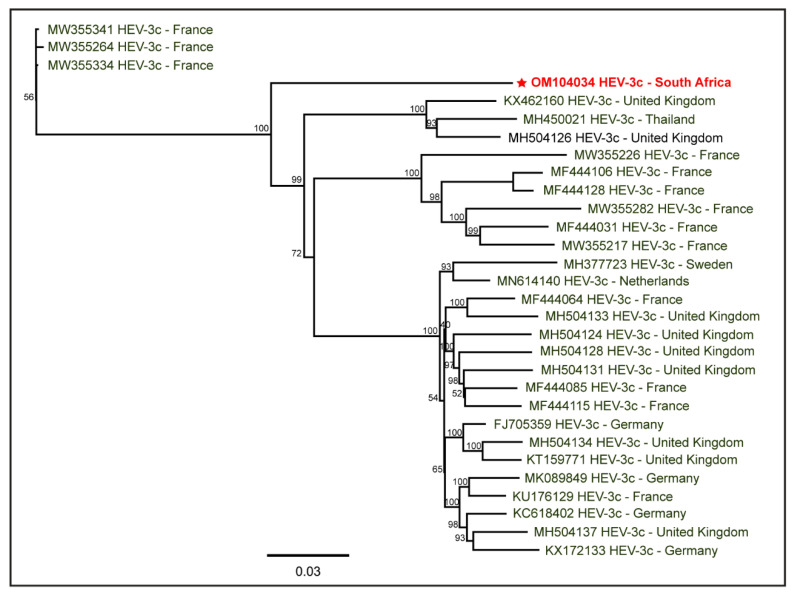
The PhyML tree of full-length genomes of HEV-3c reported from various geographical locations. The South African swine HEV-3c genome is highlighted in red. More HEV-3c genomes from other geographical locations, including South Africa, may be required for a conclusive epidemiological determination of the South African swine HEV-3c genome characterized in this study.

To determine the epidemiology of the South African swine HEV-3c genome, we used the available full-length HEV-3c genomes reported from different hosts worldwide. The PhyML tree (Figure 4) depicts the relatedness of the South African swine HEV-3c genome with other reported HEV-3c genomes. Due to the existing gap in the availability of the HEV-3c genome sequences from various geographical locations, including South Africa, the epidemiology of the South African swine HEV-3c genome characterized in this study remains inconclusive. While HEV-3c has been detected in domestic pig herds, porcine liver samples, and other pork products in various countries, including Brazil, Germany, Italy, and the Netherlands, they generated only partial sequences of ORF1 and/or ORF2, upto 476 nt; therefore, the current full-length HEV-3c genomes available in the NCBI-GenBank are only from humans and wild boars.

In summary, the present study using phylogenetics and ‘HEVnet Typing Tool’ characterized and reported a near full-length HEV-3c genome generated from the domestic pigs raised at a backyard farm in the uMgungundlovu District of the Kwa-Zulu-Natal province, South Africa. To our knowledge, this is the first near full-length HEV-3c genome generated from the naturally infected domestic pigs in South Africa. More full-length HEV-3c genomes originating from various hosts and geographical locations, including South Africa, would be required for a conclusive determination of the epidemiology of the swine HEV-3c genome characterized in this study.

## 4. Discussion

The HEV-3 genotype currently has 14 defined subtypes: 3a, 3b, 3c, 3d, 3e, 3f, 3g, 3h, 3i, 3j, 3k, 3l, 3m, and 3ra [64]. Pierini et al. (2021) generated four novel full-length HEV genomes from the liver samples collected from hunted wild boars in Central Italy during 2016–2020. Notably, these HEV strains were divergent from the reported and classified HEV-3 strains in any subtype defined thus far, and therefore, were tentatively named HEV-3n subtype [67]. This indicates that increased HEV surveillance in Suidae might further expand the diversity of HEV-3 subtypes.

We, for the first time, generated and characterized a near full-length genome of HEV-3c from a pooled saliva sample collected from domestic pigs at a backyard farm in the uMgungundlovu District of the KwaZulu-Natal province, South Africa. Previously, HEV-3c has been detected in humans [68,69,70], wild boars [67,71,72,73], and domestic pigs [39,71,72,74,75] or porcine livers and pork products [33,68,75,76] in various countries. Rutjes et al. (2009) detected HEV-3c RNA in the farmed domestic pigs and one porcine liver sample purchased from the butcher shop in the Netherlands. In addition, they also detected HEV-3e RNA in the porcine liver sample and suggested the possibility of foodborne HEV transmission to the human populations in the Netherlands [72]. Wenzel et al. (2011) detected HEV-3a and HEV-3c RNA in porcine liver samples purchased from butcher shops and grocery stores in Regensburg, Germany. They reported that the swine HEV-3a and HEV-3c sequences had high sequence homology to the HEV isolates previously reported from the patients with acute HEV infections in the same geographic region and suggested that consuming undercooked pork meat may be the most probable cause of HEV zoonotic transmission in the study area [33]. In another study, de Souza et al. (2012) detected HEV-3c RNA in pig liver samples collected from slaughtered pigs in Brazil [75]. More recently, Chelli et al. (2021) detected HEV-3c RNA in three liver samples collected from October 2017 to July 2019 from slaughtered pigs in Italy. These three pig liver samples originated from the pigs that were imported from other European Union countries to Italy for slaughtering. Notably, the three partial HEV-3c genomes generated were closely related to the HEV genome sequences reported from the human populations in previous years in the United Kingdom and France [39]. Sabato et al. (2020) studied the datasets of all HEV-3 genomes generated from humans, wild boars, and domestic pigs in Italy until June 2019 and suggested that HEV-3f was the most frequently reported subtype from humans and pigs in Italy, followed by the HEV-3e and HEV-3c [77]. These data suggest that domestic pigs can serve as a host (and reservoir) for HEV-3c viral infections.

In South Africa, several studies have reported HEV seroprevalence in human populations. For example, Madden et al. (2016) conducted a sero-surveillance in patients visiting a hospital in the Western Cape, South Africa, and determined a high HEV seroprevalence (anti-HEV IgG; 27.9%) in patients ≥ 30 years of age with no liver disease. Since the patients investigated were not exposed to pigs, foodborne transmission due to the consumption of pork meat was suspected [78]. They also reported a clinical case of HEV-3e infection in a 54-year-old male who was admitted to the hospital due to acute liver failure and later died on day 3 of admission in the hospital. The IgM test performed on day 3 (the day he demised) returned positive, and the subsequent PCR test detected HEV-3e RNA [78]. Korsman et al. (2019) detected a comparable anti-HEV IgG seroprevalence (29.5%); however, the anti-HEV IgM seroprevalence was 1.6% in the sera samples of the hospitalized patients with acute hepatitis in Cape Town. The anti-HEV IgM positive sera samples were PCR negative for the HEV RNA [79] and therefore did not report on the HEV subtypes. In another study, Korsman et al. (2019) detected HEV RNA in 2 of the 144 porcine liver spread samples purchased from butcheries and supermarkets in Cape Town. One partial HEV capsid protein sequence comprising 304 nucleotides (GenBank accession: MF503296), generated from one of these pork livers, belonged to HEV-3e and was closely related to the other HEV genomes reported from the humans in Cape Town [76]. 

While there is an existing gap in the information on circulating HEV subtypes in South African human and domestic pig populations, the available limited data suggest that HEV-3e has been sporadically detected previously in the porcine liver spread samples and in one clinical case as stated above. Our study, for the first time, reported a near full-length genome of HEV-3c from naturally infected domestic pigs raised on a South African backyard farm. Large-scale molecular and genomic surveillance in South African domestic pig populations would be useful to detect the prevalence of HEV RNA and identify the circulating HEV subtypes to explore which HEV subtypes might be entering the South African food chain via pork meat consumption. As numerous studies from Europe have suggested the foodborne transmission of HEV-3c to humans, we recommend that pork consumers should properly cook pork meat to avoid the possibility of zoonotic transmission of HEV. 

## 5. Conclusions

To our knowledge, this is the first study to characterize and report the near full-length genome of HEV subtype 3c from naturally infected backyard pigs in South Africa. We recommend conducting nationwide molecular and genomic surveillance to realize HEV prevalence in backyard pigs and to identify circulating HEV subtypes to evaluate the zoonosis risk in South Africa. 

## Data Availability

The HEV genome characterized in this study has been deposited into the NCBI-GenBank (accession number: OM104034).
